# Notes and News

**Published:** 1905-10-14

**Authors:** 


### Notes and News.

The Japanese School of Ju-Jitsu at 305 Oxford Street, promises us a work on Ju-Jitsu in the near future. It will be of a practical nature, following the lines of instruction carried out at the school.

It is stated on authority that the Barnardo Homes have an indebtedness of no less than £249,000, and that of this £22,000 is due to the bank. On the other hand, it appears that the organisation possesses property valued at fully the whole of this amount. Lord Brassey suggests that the nation should remove the debt as a fitting memorial to Dr. Barnardo.

The International Congress on the Education and Protection of Children at Liège seems to have been somewhat marred by an over-abundance of material and too short a time for the consideration of it. The meeting was very representative, but as there is more theory than practice in connection with its subject, it is not easy to draw conclusions, nor can it be said that the communications had much definite importance.

A Fancy Fair has recently been held in Bridgewater in aid of the Infirmary. It has resulted in a contribution of £1,200 for the Institution. There is no doubt that whilst in London it is very doubtful whether these entertainments do not do more harm than good to a charity, in the provinces they are useful in every way. They provide occupation and entertainment for the promoters and patrons, and stimulate local interest towards the institution to be benefited, and are also not unprofitable to local tradesmen.

Whilst in England and Germany the problem of the housing of the working classes is receiving close attention, in urban Italy the matter has become very serious, without apparently any action having been taken either by the Government or municipalities. In Italy there are not many rich and charitable persons to lead the way, as was the case in England, where private enterprise, in the form of the Rowton Houses, set an example which fortunately public authorities are now following.

The Swedish Government have declared the Prussian provinces of East Prussia and Posen and the Government district of Stettin to be infected by cholera, and the Norwegian authorities have added Posen, East Prussia, and Poland to the list of places declared to be infected by cholera. A message from Constantinople says, "Free pratique granted to arrivals from Aden, Port Said, and Damietta; twenty-four hours' observation with disinfection and rat destruction imposed on arrivals from Alexandria."

At the Tuberculosis Congress in Paris Dr. Heron, in drawing attention to the importance to the nation of the teaching of the principles of hygiene in schools, recommended that the instruction should include the chief needs of the body for the maintenance of health, the hygiene of clothing and of practical physical exercises, the outline of food preparation, the nutritive and other values of food, selection and cooking of inexpensive foods, and the elements of house management and hygiene.

At Charing Cross Hospital the following Entrance Scholarships have been awarded: The Epsom Scholarship (100 guineas) to Mr. E. G. H. Cowen, the Livingstone Scholarship (100 guineas) to Mr. N. H. Harrison, the Huxley Scholarship (55 guineas) to Mr. M. A. Farr, Universities Scholarships (each 72 guineas) to Mr. F. S. Poole and Mr. D. A. Powell. An Entrance Scholarship of 40 guineas has also been awarded to Mr. M. B. Bayley, and Universities Exhibitions of 36 guineas each to Mr. A. V. Poyser and Mr. J. F. Hornsey.

Whilst the public and charitable institutions alike are so careless it is not surprising that persons—Alfred Rogers and his wife, who have recently been imprisoned for fraud—should succumb to temptation. The Metropolitan and Provincial Association for Supplying Free Meals to Poor Children apparently delivered one of their collecting-boxes to the man, who, with the assistance of his wife, used it to obtain money for himself. No amount of warning seems effectual in preventing the public from giving to all and sundry persons who beg, and associations are equally irresponsible in their methods.

It is interesting to note how frequently Japanese methods are now quoted as examples of what should be done, often on the most scientific basis. Quite recently a member of the Education Committee of the Glamorgan County Council pointed out that Japanese children were not allowed to attend school until after six years of age, as the Japanese believe that science has conclusively proved that school education before six is physiologically and mentally detrimental. In England the poor mothers very much resent the exclusion of the young children from school, as it curtails their earning powers if the child remains at home. If school nurseries were established they might not only satisfy the mothers, but also create a hygienic supervision of the children at an age when it would be of great importance.

